# The potential of epigenetic therapies in neurodegenerative diseases

**DOI:** 10.3389/fgene.2014.00220

**Published:** 2014-07-14

**Authors:** Fabio Coppedè

**Affiliations:** Department of Translational Research and New Technologies in Medicine and Surgery, University of PisaPisa, Italy

**Keywords:** Alzheimer's disease, Parkinson's disease, amyotrophic lateral sclerosis, Huntington's disease, DNA methylation, histone tail modifications, histone deacetylase inhibitors (HDACi), therapeutic approaches

## Abstract

Available treatments for neurodegenerative diseases such as Alzheimer's disease, Parkinson's disease, amyotrophic lateral sclerosis, and Huntington's disease, do not arrest disease progression but mainly help keeping patients from getting worse for a limited period of time. Increasing evidence suggests that epigenetic mechanisms such as DNA methylation and histone tail modifications are dynamically regulated in neurons and play a fundamental role in learning and memory processes. In addition, both global and gene-specific epigenetic changes and deregulated expression of the writer and eraser proteins of epigenetic marks are believed to contribute to the onset and progression of neurodegeneration. Studies in animal models of neurodegenerative diseases have highlighted the potential role of epigenetic drugs, including inhibitors of histone deacetylases and methyl donor compounds, in ameliorating the cognitive symptoms and preventing or delaying the motor symptoms of the disease, thereby opening the way for a potential application in human pathology.

## Introduction

Epigenetic processes, including DNA methylation and post-translational modifications of the histone tails, influence gene expression levels without involving changes of the primary DNA sequence, and play a fundamental role in embryonic development, cell differentiation, and maintenance of the cellular identity (Martín-Subero, 2011). They are also dynamically regulated in neurons in response to experiential stimuli to regulate the expression of memory-related genes, and play a role in the formation, consolidation and storage of memory (Guo et al., 2011a; Puckett and Lubin, 2011; Sultan and Day, 2011; Lattal and Wood, 2013). Therefore, it is not surprising that changes of epigenetic marks might be at least partially involved in the onset and progression of neurodegenerative diseases (Coppedè, 2014), leading researchers to hypothesize that targeting the epigenome with compounds exerting epigenetic properties could represent a promising preventative and therapeutic approach for neurodegeneration (Karagiannis and Ververis, 2012; Harrison and Dexter, 2013).

## DNA methylation

DNA methylation typically occurs in CpG islands (regions unusually enriched in the CpG dinucleotide, found in the promoter region of almost a half of human genes and relatively absent from most other places in the genome) and consists of the addition of a methyl group (CH3) to the 5′ position of the cytosine pyrimidine ring, forming 5-methylcytosine (5-mC). The reaction is mediated by DNA methyltransferases (DNMTs) using S-adenosylmethionine (SAM) as the methyl donor compound. Methylation of CpG islands in the promoter region of a gene can be specifically recognized by a set of proteins called methyl-CpG binding proteins (MBPs), that contain a transcription repression domain to interact with other proteins and enhance DNA methylation-mediated transcriptional repression (Fournier et al., 2012; Jones, 2012). Enzymes of the TET (ten-eleven translocation) family promote DNA demethylation converting 5-methylcytosine to 5-hydroxymethylcytosine (5-hmc), a modified form of cytosine hydroxymethylated at the 5′ position showing lower affinity for MBPs and present at high levels in the brain (Guo et al., 2011b).

## Histone tail modifications

The nucleosome is the structural and functional unit of chromatin and consists of approximately 147 DNA base pairs wrapped twice around a histone octamer consisting of two copies each of the core histones H2A, H2B, H3, and H4. Nucleosomes are connected by stretches of “linker DNA” and linker histones, such as histone H1, and are involved in chromatin compaction (Luger et al., 1997). Histone tail modifications include acetylation, methylation, phosphorylation, ubiquitylation, sumoylation and other post-translational modifications (Berger, 2007). The combination of those modifications, and their crosstalk with DNA methylation and chromatin remodeling proteins, regulate the chromatin structure in a dynamic fashion. Indeed, chromatin can exist in a condensate inactive state (heterochromatin) or in a non-condensate and transcriptionally active state (euchromatin) (Berger, 2007).

Acetylation and methylation of the histone tails represent the two most studied mechanisms of histone modifications. Acetylation of lysine residues on histone tails neutralizes their positive charge and decreases their affinity for the DNA, leading to an increased accessibility of transcriptional regulatory proteins to chromatin templates. Histone acetyltransferases (HATs) and deacetylases (HDACs) catalyze lysine acetylation and deacetylation, respectively (Berger, 2007). Eighteen HDAC enzymes are known in mammals which are divided into four classes (I-IV). A summary of their function and localization is provided in Table 1, more detailed information can be found in recent research and review articles (Broide et al., 2007; Zakhary et al., 2010; Joshi et al., 2013; Lazo-Gómez et al., 2013). Methylation on either lysine or arginine residues of the histone tails can be associated with either condensation or relaxation of the chromatin structure, since several sites for methylation are present on each tail thus allowing different combinations (Martin and Zhang, 2005). Crosstalk between acetylation and methylation at specific residues further influences transcriptional activation, for example di- and tri-methylation of H3K4 (H3K4me2 or H3K4me3), and acetylation at H3/H4 (H3K9Ac and H4K9Ac) are generally linked to an open chromatin structure that allows gene transcription, while histone hypoacetylation, and tri-methylation at H3K9 (H3K9me3) and H3K27 (H3K27me3) are considered repressive marks (Berger, 2007). Histone methyltransferases (HMTs) transfer the methyl group from SAM to histone tail residues, and histone demethylases (HDMTs) are the “erasers” of that mark (Martin and Zhang, 2005).

**Table 1:** **Mammalian histone deacetylase (HDAC) proteins and some of their inhibitors (HDACi)**.

**Histone deacetylase (HDAC) class/function**	Proteins/sub-cellular localization/brain and spinal cord regions of expression^*^	**HDAC inhibitors (HDACi)**
Class I (Zinc dependent)	HDAC 1/nucleus/Ce, Ob, A, Hi, Co, Ch, P, Sc, Hy, Sn-c, Sn-r, M, Na, Cp	Pan-inhibitors: trichostatin A, vorinostat, butyrate, phenylbutyrate, valproate
Involved in: regulation of gene-specific transcription through the formation of stable transcriptional complexes	HDAC 2/nucleus/Hi, Ce, Ob, Co, A, CP, Na, P, Sc, Sn-c, Hy, M, Sn-r	Selective inhibitors: MS-275 (HDAC1), 4b (HDAC1 and 3)
	HDAC 3/shuttle between nucleus and cytoplasm/Hi, Ce, Ob, Co, A, Sn-c, Hy, Cp, Na, Ch, M, Sc, P, Sn-r, Gp	
	HDAC 8/nucleus / Hi, Ce, Ob, A, Sn-c, Co, Hy, P, M, Sc	
Class IIa (Zinc dependent)	HDAC 4/nucleus-cytoplasm/Ce, Hi, Ob, Co, A, Sn-c, Hy, P, M, Sc, Sn-r, Na, Cp, Gp	Pan-inhibitors: trichostatin A, vorinostat, butyrate, phenylbutyrate, valproate
Shuttle between nucleus and cytoplasm to interact with both nuclear and cytoplasmic proteins, have histone deacetylase activity by interacting with HDAC3	HDAC 5/nucleus-cytoplasm/Ce, Hi, Ob, Co, Sn-c, Hy, Cp, Na, A, P, M, Sc, Ch, Sn-r, Gp	
	HDAC 7/nucleus-cytoplasm/Ce, Hi, Ob, A, Sn-c, Co, Cp, Na, Hy, P, M, Sc	
	HDAC 9/nucleus-cytoplasm/Hi, Sn-c, Ob, Co, A, P, Ce	
Class IIb (Zinc dependent)	HDAC 6/cytoplasm/Hi, Ob, Ce, Co, A, Sn-c, Hy, P, Cp, Na, M, Sc	Pan-inhibitors: Trichostatin A, vorinostat
HDAC6: take part in the microtubule network by acting on α-tubulin and tau proteins, and plays a fundamental role in the formation of protein aggregates; HDAC10: cytoplasmic deacetylase	HDAC 10/cytoplasm/Hi, Ob, Ce, A	
Class III (nicotinamide dependent)	Sirtuin (SIRT) 1 and 6/nucleus/(SIRT 1) Co, Hi, Ce, Hy, Sc	Pan-inhibitor: nicotinamide
SIRT 1-3 have robust deacetylase activity; SIRT4 is an ADP-ribosyltransferase; SIRT 5 has deacetylase and other activities; SIRT 6 has ADP-ribosyl transferase and deacetylase activities; SIRT 7 is a deacetylase	SIRT 2/cytosol	Selective inhibitors: AK-1, AGK-2, AK-7 (sirtuin 2)
	SIRT 3, 4, 5/mitochondria	
	SIRT 7/nucleolus	
Class IV (Zinc dependent)	HDAC 11/nucleus/Hy, A, Ob, P, Co, Cp, Na, Hi, Sn-c, Sc, M, Sn-r, Gp	LAQ824
Member of the survival of motor neuron complex, has a functional role in mRNA splicing		

* Expression levels are obtained from studies in brain regions of rodents (Broide et al., 2007; Zakhary et al., 2010) and ordered from high expression (first) to low expression (latter). A, amygdala; Ce, cerebellum; Ch, choroid plexus; Co, cortex; Cp, caudate putamen; Gp, globus pallidus; Hi, hippocampus; Hy, hypothalamus; Na, nucleus accumbens; M, medulla; Ob, olfactory bulb; P, pons; Sc, spinal cord; Sn-c, Substantia nigra pars compacta; Sn-r, Substantia nigra pars reticulata.

## Alzheimer's disease

Alzheimer's disease (AD) is the most common neurodegenerative disorder and the primary form of dementia in the elderly. The disease symptoms get worse over time and available treatments may only help keeping patients from getting worse for a short period. Affected brain regions show extracellular amyloid deposits called senile plaques (SP) and intra-neuronal aggregates of hyperphosphorylated tau protein, called neurofibrillary tangles (NFT). The primary component of SP is the amyloid β (Aβ) peptide, resulting from the proteolytic cleavage of its precursor protein (APP), mediated by the proteins β-secretase (BACE1) and γ-secretase, a protein complex composed of presenilins and other proteins (Overk and Masliah, 2014). Mutations in *APP* and presenilin (*PSEN1* and *PSEN2*) genes cause early-onset (<65 years) AD, overall accounting for almost 1% of the cases. However, most of AD (95–98%) are late-onset (>65 years) sporadic forms, resulting from complex interactions among genetic, environmental and stochastic factors superimposed on the physiological decline of cognitive functions with age (Coppedè, 2014). Epigenetic mechanisms are at the core of gene-environment interactions and believed to play a role in AD (Migliore and Coppedè, 2009).

Particularly, DNA methylation changes in AD are supported by studies in cell cultures and animal models (reviewed in Coppedè, 2014), by a number of studies that have shown reduced global levels of 5-mC and 5-hmC in human post-mortem AD brains (Mastroeni et al., 2010; Chouliaras et al., 2013), and by recent epigenome-wide approaches searching for candidate genes (Bakulski et al., 2012; Sanchez-Mut et al., 2014). Also changes in histone tail modifications have been observed in post-mortem AD brains, including decreased levels of H3 acetylation in the temporal lobe (Zhang et al., 2012), and increased levels of the histone deacetylases HDAC6 and HDAC2, the first leading to increased tau phosphorylation and the latter repressing genes required for learning and memory (Ding et al., 2008; Gräff et al., 2012). However, studies in humans are scarce, limited by the small sample size of case and control brains, and often conflicting (Coppedè, 2014; Coppieters et al., 2014). Those performed with rodents and cell cultures revealed the contribution of histone modifications to learning and memory processes, and the manipulation of histone acetylation with HDAC inhibitors (HDACi) in AD animal models often resulted in both prevention of cognitive deficits and memory recovery (Karagiannis and Ververis, 2012).

### Epigenetic interventions in alzheimer's disease

Folate, other B group vitamins (B_2_, B_6_, and B_12_), and homocysteine (hcy), participate in one-carbon metabolism, the metabolic pathway required for SAM production, and there is indication of impaired one-carbon metabolism and reduced DNA methylation potential in AD individuals (Coppedè, 2010). Human neuroblastoma cells and transgenic AD mice maintained under conditions of B vitamin deficiency showed *PSEN1* promoter demethylation, with subsequent increased production of presenilin1, BACE1 and APP proteins, and Aβ deposition in the animal brains. By contrast, SAM supplementation induced an opposite tendency, restored *PSEN1* methylation levels, and reduced the progression of the AD-like features induced by B vitamin deficiency in mice (Fuso et al., 2005, 2008, 2012). There is some indication from elderly human subjects suggesting that B vitamin supplementation can slow the atrophy of specific brain regions that are associated with cognitive decline, but additional trials are warranted to see if the progression to dementia can be prevented (Douaud et al., 2013). In addition, dietary SAM supplementation reduced oxidative stress (Tchantchou et al., 2008) and delayed Aβ and tau pathology in transgenic AD mice (Lee et al., 2012), suggesting a possible role of SAM as a neuroprotective dietary supplement in AD.

Another line of active research involves the manipulation of histone acetylation with HDACi in AD animal models (Karagiannis and Ververis, 2012). Several inhibitors of those enzymes have been developed, including valproic acid, trichostatin A, sodium phenylbutyrate, and vorinostat that interact with zinc-dependent HDAC proteins (class I, class II, and class IV), nicotinamide that inhibits class III HDACs, and more recent compounds that selectively inhibit certain HDACs (Table 1). Early studies in the field revealed that sodium butyrate administration for 4 weeks was able to reinstate learning and memory in transgenic mice that already suffered from severe AD pathology (Fischer et al., 2007). Subsequent studies revealed that sodium butyrate injection in a transgenic mouse model of AD is correlated with decreased tau phosphorylation and restoration of dendritic spine density in hippocampal neurons (Ricobaraza et al., 2010), and prolonged treatment in a transgenic mouse model for amyloid deposition (APP/PS1 mice) improved associative memory by increasing both hippocampal histone acetylation and the expression of genes implicated in associative learning (Govindarajan et al., 2011). Others showed that after fear conditioning training, the levels of hippocampal acetylated histone 4 (H4) in APP/PS1 mice were about 50% lower than in wild-type littermates. However, an acute treatment with trichostatin A prior to training rescued both acetylated H4 levels and contextual freezing performances to wild-type values (Francis et al., 2009). Several similar examples are available in the literature concerning HDACi and memory function in AD animal models, for example 2–3 weeks treatment with either sodium valproate, sodium butyrate, or vorinostat reversed contextual memory deficits in APP/PS1 mice (Kilgore et al., 2010), a 10 days treatment with entinostat, a selective inhibitor of HDAC1, reduced neuroinflammation and amyloid plaque deposition and improved behavioral impairment in APPPS1-21 mice (Zhang and Schluesener, 2013), and a 4 week treatment with a class II inhibitor in transgenic AD mice over-expressing mutant APP, presenilin1 and tau proteins (3 × AD mice), improved memory functions and decreased Aβ and phosphorylated tau levels (Sung et al., 2013). Taken overall, those studies suggest that targeting histone modifications with HDACi can improve cognition and reduce AD-like features in AD models (Table 2).

**Table 2:** **Some examples of the effects of histone deacetylase inhibitors (HDACi) in animal models of neurodegenerative diseases**.

**Compound**	Experimental model^*^	**Results**	**References**
Sodium butyrate	Transgenic AD mice (CK-p25)	4 week intra-peritoneal administration improved learning and memory	Fischer et al., 2007
Trichostatin A	Transgenic AD mice (APP/PS1)	Acute treatment prior to fear conditioning training rescued hippocampal H4 acetylation levels and contextual freezing performances	Francis et al., 2009
Sodium butyrate, sodium valproate, or vorinostat	Transgenic AD mice (APP/PS1)	2–3 weeks intra-peritoneal injection reversed contextual memory deficits	Kilgore et al., 2010
Sodium butyrate	Transgenic AD mice (APP/PS1)	Chronic intra-peritoneal injection improved associative memory	Govindarajan et al., 2011
Sodium butyrate	Transgenic AD mice (Tg2576)	5 week intra-peritoneal injection decreased tau phosphorylation and restored dendritic spine density in hippocampal neurons	Ricobaraza et al., 2010
MS-275 (entinostat)	Transgenic AD mice (APP/PS1)	10 days oral administration ameliorated neuroinflammation and cerebral amyloidosis and improved behavior	Zhang and Schluesener, 2013
W2 (Class II HDACi)	Transgenic AD mice (3 × AD)	4 weeks intra-peritoneal injection improved memory functions and decreased Aβ and phosphorylated tau levels	Sung et al., 2013
Sodium butyrate or vorinostat	*Drosophila* model of PD overexpressing α-synuclein	20 days treatment reduced α-synuclein mediated toxicity	Kontopoulos et al., 2006
AK-1 or AGK-2 (sirtuin 2 HDACi)	*Drosophila* model of PD overexpressing α-synuclein	20 days treatment reduced α-synuclein mediated toxicity	Outeiro et al., 2007
Valproic acid	Rotenone-induced PD rat model	4 weeks oral administration counteracted α-synuclein nuclear translocation and toxicity	Monti et al., 2010
Sodium butyrate	MPTP-induced PD mouse model	14 days oral administration up-regulated DJ-1 expression and reduced neurotoxicity	Zhou et al., 2011
Sodium butyrate	Rat model of PD	5 days intra-peritoneal injection alleviated cognitive deficits	Rane et al., 2012
Sodium butyrate	Rotenone-induced PD fly model	3 days oral administration improved locomotor impairment and early mortality	St Laurent et al., 2013
Sodium butyrate, trichostatin A, or valproate	ALS mice (SOD1-G93A)	Several studies show that treatments with one ot those agents delayed disease progression and/or increased animal survival	Sugai et al., 2004; Ryu et al., 2005; Yoo and Ko, 2011
4b (HDAC1i and HDAC3i)	Transgenic HD mice	10–12 weeks injections improved motor functions and elicited cognitive decline	Jia et al., 2012
AK-7 (sirtuin 2 HDACi)	Transgenic HD mice	4 weeks intra-peritoneal injection improved motor functions, extended survival and reduced mutant huntingtin aggregation	Chopra et al., 2012

* Most of the used transgenic models over-expressed mutant proteins (amyloid precursor protein and presenilin 1 in AD APP/PS1 models, α-synuclein in PD models, mutant superoxide dismutase “SOD1” in ALS models, and mutant huntingtin in HD model) or were induced by pesticide exposure (such as rotenone or 1-methyl-4-phenyl-1,2,3,6-tetrahydropyridine “MPTP”). AD, Alzheimer's disease; ALS, amyotrophic lateral sclerosis; HD, Huntington's disease; PD, Parkinson's disease).

## Parkinson's disease

Parkinson's disease (PD), the second most common neurodegenerative disorder after AD, is clinically characterized by resting tremor, rigidity, bradykinesia, and postural instability as well as non-motor symptoms such as autonomic insufficiency, cognitive impairment, and sleep disorders. Pathologically, PD is characterized by progressive and profound loss of neuromelanin containing dopaminergic neurons in the *substantia nigra* with the presence of eosinophilic, intracytoplasmic inclusions called Lewy bodies (LBs; containing aggregates of α-synuclein and other substances), and Lewy neurites in surviving neurons. Although some improvements of the symptoms can be achieved with levodopa and dopaminergic therapy, there is no available treatment to halt disease progression (Thomas and Beal, 2011). The majority of PD cases are sporadic, likely arising from a combination of polygenic inheritance, environmental exposures, and complex gene-environment interactions superimposed on slow and sustained neuronal dysfunction due to aging (Migliore and Coppedè, 2009). In a minority of the cases PD is inherited as a Mendelian trait, and studies in PD families allowed the identification of at least 15 PD loci (PARK1-15) and several causative genes (Nuytemans et al., 2010). Most of the studies evaluating the role of epigenetics in disease pathogenesis have focused on the analysis of promoter methylation of causative PD genes in post-mortem brains and peripheral blood cells of affected individuals (Coppedè, 2012). Particularly, reduced methylation levels of the gene coding for α-synuclein (*SNCA* gene), the first identified causative PD gene, were observed in several brain regions of sporadic PD patients including the *substantia nigra* (Jowaed et al., 2010; Matsumoto et al., 2010). In addition, it was shown that α-synuclein sequesters DNMT1 from the nucleus into the cytoplasm, and reduced DNMT1 levels were observed in post-mortem PD brains (Desplats et al., 2011).

Our knowledge of histone modifications in PD derives mainly from studies in cell cultures and animal models of the disease, such as those induced by the mitochondrial toxins 1-methyl-4-phenylpyridinium (MPP+), paraquat and rotenone, or those overexpressing human α-synuclein (Harrison and Dexter, 2013). Overall, those studies revealed that α-synuclein interacts with histones and inhibits histone acetylation, and that several HDACi are neuroprotective against α-synuclein-mediated toxicity (Table 2).

### Epigenetic interventions in parkinson's disease

The analysis of nigral neurons of mice exposed to the herbicide paraquat revealed that α-synuclein translocates into the nucleus and binds to histones (Goers et al., 2003). Studies in *Drosophila* showed that α-synuclein mediates neurotoxicity in the nucleus, binds directly to histone H3, and inhibits histone acetylation. The toxicity of α-synuclein was rescued by the administration of sodium butyrate or vorinostat (Kontopoulos et al., 2006). Selective inhibition of the histone deacetylase sirtuin 2 rescued α-synuclein-mediated toxicity in several models of PD (Outeiro et al., 2007). In addition, valproic acid resulted neuroprotective in a rotenone-induced rat model of PD counteracting α-synuclein translocation into the nuclei (Monti et al., 2010). Neurotoxic pesticides and paraquat were shown to increase histone acetylation in mice brains or cell culture models (Song et al., 2010, 2011), and an increasing number of studies reveal protective effects of HDACi on neurons following a neurotoxic-induced insult (Harrison and Dexter, 2013). For example, it was recently shown that trichostatin A selectively rescues mitochondrial fragmentation and cell death induced by MPP+ in human neuroblastoma cells (Zhu et al., 2014), and that sodium butyrate improves locomotor impairment and early mortality in a rotenone-induced *Drosophila* model of PD (St Laurent et al., 2013), alleviates cognitive deficits in a rat model of PD in the pre-motor deficit stage (Rane et al., 2012), and up-regulates DJ-1 protein expression and protects neurons in cell cultures and mouse models against MPP+ toxicity (Zhou et al., 2011). DJ-1 is involved in the protection of oxidative stress, and mutation of *DJ-1* gene cause early-onset PD (Bonifati et al., 2003).

All the above studies suggest that histone modifications occur in α-synuclein-mediated as well as environmentally-induced dopaminergic neuronal cell death and suggest a protective role for HDACi in those models (Table 2).

## Other neurodegenerative diseases

Several similar examples of the potential neuro-protective effects obtained by targeting the epigenome are available for other neurodegenerative diseases than AD or PD. For example, SAM supplementation in a transgenic mouse model (SOD1-G93A) of amyotrophic lateral sclerosis (ALS) delayed the onset of motor neuron pathology (Suchy et al., 2010). Also HDACi ameliorated disease progression in ALS animal models. For example, sodium phenylbutyrate significantly extended survival in G93A transgenic ALS mice (Ryu et al., 2005). The combination of phenylbutyrate and riluzole ameliorated gross lumbar and ventral horn atrophy, attenuated lumbar ventral horn neuronal cell death, and decreased reactive astrogliosis in ALS G93A mice (Del Signore et al., 2009). Similarly, combined lithium and valproate treatment delayed disease onset, reduced neurological deficits and prolonged survival (Feng et al., 2008), and treatment with trichostatin A (Yoo and Ko, 2011), or valproate (Sugai et al., 2004), delayed disease progression and/or increased survival in the SOD1-G93A mice. A phase II study in ALS individuals revealed that sodium phenylbutyrate was safe and tolerable, and histone acetylation was significantly increased after sodium phenylbutyrate administration (Cudkowicz et al., 2009). Conversely, a trial using valproic acid did not show a beneficial effect on survival or disease progression in patients with ALS (Piepers et al., 2009).

Research in Huntington's disease (HD), a neurodegenerative diseases caused by trinucleotide repeat expansion in the gene (*HTT*) coding for the huntingtin protein, revealed that mutant huntingtin directly interacts with HAT proteins, leading to altered histone acetylation (Steffan et al., 2000; Jiang et al., 2006). Numerous studies revealed that treatment with HDACi arrested the ongoing progressive neuronal degeneration in both fly and mouse models of HD (reviewed by Gray, 2010). Some recent examples using selective HDACi are listed in Table 2 (Chopra et al., 2012; Jia et al., 2012), and other compounds are under investigation (Bürli et al., 2013; Duan, 2013).

## Concluding remarks

The studies described in this manuscript provided evidence that targeting the epigenome, mainly with small drugs such as HDACi that are able to cross the blood brain barrier, delayed the onset and progression of the symptoms in animal models of neurodegenerative disease (Table 2). Several other examples are available in the literature, but cannot be described in details due to limited space allowance.

Despite the encouraging results obtained so far in animal models, highlighting the potential of HDACi for the treatment of neurodegenerative diseases, additional research is required prior to their application to humans. Indeed, as suggested by many authors, reasons of concern include the multi-targeted and multi-cellular effects exerted by most of those drugs, the possibility of unexpected side effects, and the anatomical and metabolic differences between humans and rodents, suggesting that further investigation is warranted to clarify the most appropriate therapeutical approaches, including the use of pan-HDACi or selective inhibitors, the timing, the dosage regimen, a better understanding of the interplay among histone tail modifications and other mechanisms that regulate gene expression, and the evaluation of potential side effects (Harrison and Dexter, 2013; Fischer, 2014). In addition, the mode of delivery varied among studies, and not all HDACs are similarly expressed in the brain regions affected by AD, PD, or other neurodegenerative diseases (Table 2). Indeed, albeit HDAC2 and HDAC6 could represent promising drug targets in AD, it remains unknown which drug or dosing regime present the most efficacy, and similar conclusions can be drawn for other neurodegenerative diseases (Harrison and Dexter, 2013; Fischer, 2014). Moreover, not all drugs always work. For example, valproic acid treatment in the SOD1 transgenic ALS mice only prevented motor neuron death, but failed to improve survival or motor performance (Rouaux et al., 2007), and similar results were observed in the clinical trial in humans (Piepers et al., 2009). Another limitation to our knowledge is that drugs have been tested in animal models bearing mutations in human genes, such as *SOD1*, that only account for only about 1–2% of the total human cases (Table 2). Moreover, other writer or eraser proteins of the histone code than HDACs could represent potential targets for drug development, but studies are scarce (Fischer, 2014), and natural compounds found in the diet, including folate, vitamins, polyphenols, and flavonoids, alter the availability of methyl groups and influence the activity of DNMTs, thereby representing potential “epigenetic” preventive factors for neurodegeneration (Coppedè, 2014).

### Conflict of interest statement

The author declares that the research was conducted in the absence of any commercial or financial relationships that could be construed as a potential conflict of interest.
